# Activation of Automatic Tube Compensation Mode Attenuates Auto‐PEEP in Chronic Obstructive Pulmonary Disease Patients

**DOI:** 10.1111/crj.70028

**Published:** 2024-10-22

**Authors:** Omid Moradi Moghaddam, Shahab Mohammadi, Mohsen Sedighi, Alireza Amanollahi, Behrooz Zaman, Mahzad Alimian, Mansoor Soltani, Mohammad Niakan Lahiji

**Affiliations:** ^1^ Trauma and Injury Research Center Iran University of Medical Sciences Tehran Iran; ^2^ Department of Critical Care Medicine, School of Medicine Iran University of Medical Sciences Tehran Iran; ^3^ Pain Research Center, Department of Anesthesiology and Pain Medicine Iran University of Medical Sciences Tehran Iran; ^4^ Department of CVD Research Center Birjand University of Medical Sciences Birjand Iran

**Keywords:** automatic tube compensation, auto‐PEEP, COPD, mechanical ventilation

## Abstract

**Introduction:**

Dynamic hyperinflation in chronic obstructive pulmonary disease (COPD) results in intrinsic positive end‐expiratory pressure (auto‐PEEP). Automatic tube compensation (ATC) is used to increase airway pressure in COPD and overcome endotracheal tube (ETT)–imposed respiratory workload. We aim to investigate effects of ATC activation on auto‐PEEP decrease in COPD.

**Methods:**

ATC was activated three times a day (1 min duration) in the morning, evening, and night shift. Auto‐PEEP was measured for the 1 min period (every 6 s) following ATC activation. Linear mixed model (LMM) was used to measure changes in auto‐PEEP and compare with baseline value. Age, gender, and COPD types were inserted in model as covariates and analyzed using SPSS.

**Results:**

A total of 60 patients including COPD (*n* = 40) and COPD with exacerbation (*n* = 20) were included. Compared with exacerbated COPD, baseline auto‐PEEP in COPD was significantly lower in morning (*p* = 0.011), evening (*p* = 0.043), and night shift (*p* = 0.007). After ATC activation, auto‐PEEP decreased significantly in COPD in morning, evening, and night (*p* = 0.001), but magnitude of this decrease was notably larger in COPD than in exacerbated COPD (*p* = 0.001). Moreover, there was a significant relationship between COPD exacerbation and changes in auto‐PEEP in morning (*β* = −0.27, *p* = 0.001), evening (*β* = −0.16, *p* = 0.001), and night (*β* = −0.26, *p* = 0.001).

**Conclusion:**

The activation of ATC mode in COPD patients under mechanical ventilation could decrease the value of auto‐PEEP. Nevertheless, COPD patients with an exacerbation appear to benefit less from ATC activation.

## Graphical abstract

Baseline auto‐PEEP in exacerbated form of COPD is greater than in COPD patients. ATC activation in COPD patients decreases auto‐PEEP and attenuates ETT‐related side effects. Exacerbated form of COPD limits the beneficial effects of ATC activation on minimizing dynamic hyperinflation and auto‐PEEP.

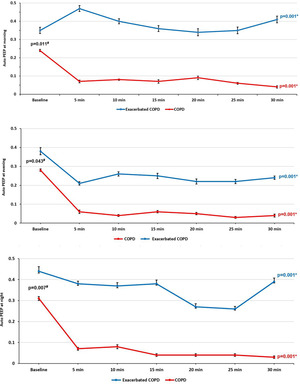

## 1 Introduction

Chronic obstructive pulmonary disease (COPD) accounts for one of the main causes of death across the world and affects millions of people [1]. Pathophysiological features of COPD include dysfunction of the small and large airways as well as the destruction of lung parenchyma and associated vasculature in highly variable combinations. COPD is characterized by a limitation of expiratory flow, which happens even during resting quiet breathing [2]. Prior investigations of selected patient populations have reported an in‐hospital mortality rate from 4% to 30% for acute exacerbation of COPD [3]. Predictors of hospitalization need in COPD patients are severe dyspnea, the presence of poor quality of life, unsatisfactory lung function (low FEV1, low PaO_2_, high PaCO_2_), the existence of pulmonary arterial hypertension, and a long‐term oxygen therapy requirement [4, 5, 6].

Mechanical ventilation (MV) is routinely used in intensive care units (ICUs) for unloading respiratory muscles and also providing appropriate gas exchange in order to allow patients time for the lung to recuperate from primary injury [7]. However, dynamic hyperinflation increases airway resistance, imposes an adverse effect on tidal ventilation, and results in intrinsic positive end‐expiratory pressure (auto‐PEEP) [8]. Most COPD patients who need invasive MV need a modest volume of inspired oxygen and a low to moderate amount of PEEP to improve oxygenation [ 9]. Nevertheless, dynamic hyperinflation and auto‐PEEP during MV in COPD patients can lead to barotrauma and hemodynamic compromise [10]. In addition, the diameter of an endotracheal tube (ETT) plays an important role in the level of respiratory resistance, and ETT narrowing increases the work of breathing, which can prolong weaning from MV and alter the breathing pattern [11].

Automatic tube compensation (ATC) is a new option that has been introduced to overcome the ETT‐imposed respiratory workload [12]. ATC mode is used to increase airway pressure by continuously measuring the drop in pressure across the ETT during inspiration, and the success rate for extubation with ATC is reported to be good [13]. Therefore, the current study is designed to investigate the effect of activating ATC mode on the auto‐PEEP changes in COPD patients who are under invasive MV.

## 2 Materials and Method

### 2.1 Study Design and Patients

This prospective investigation was performed on hospitalized COPD patients in compliance with the declaration of Helsinki and local ethics and research review committee approved the protocol of the study (IR.IUMS.REC.1400.072). COPD diagnosis for eligibility to enter the study was made through physical examination and pulmonary function tests (PFTs).

### 2.2 Inclusion and Exclusion Criteria

Inclusion criteria to enter the study were hospitalized COPD patients in ICUs who underwent invasive MV for at least 24 h, age above 18 years, and Richmond Agitation Sedation Scale (RASS) score between −5 and +1. Patients were excluded from the study if they had clogged or leaking ETT, agitation and tracheal suction during treatment, and change of ventilator setting when receiving treatment.

### 2.3 ATC Delivering

Patients were breathing through the ventilator machine (Hamilton‐C2) set on SIMV mode, tidal volume 7 cc/kg, respiratory rate 10, PEEP 5 cmH_2_O, and Fio_2_ 60%. ATC was activated three times a day at morning, evening, and night shifts for the 1‐min period. Before delivering ATC, auto‐PEEP was calculated and recorded every 6 s for a 1‐min period. Furthermore, auto‐PEEP was recorded with the same protocol after activating ATC and repeated six times while we had a 6‐min interval between times (36 min in total).

### 2.4 Statistical Analysis

IBM SPSS version 24.0 (IBM, Inc., Armonk, NY, USA) is used to perform data analysis. Continuous data are described as mean ± standard deviation (SD), and categorical data are shown as frequency (%). Linear mixed model (LMM) analysis was applied to estimate mean changes of auto‐PEEP in COPD patients over the time of the experiment. Type of COPD, age, and gender of patients were inserted in the model as covariates to assess their effects on the mean changes in auto‐PEEP. Restricted maximum likelihood with slope and intercept random effect was applied to calculate all coefficients in the model. *p* values less than 0.05 were considered significant.

## 3 Results

From 2019 to 2022, a total of 60 hospitalized COPD patients in ICUs met our criteria to enter the study. The total mean age of patients was 77.33 ± 8.29 years, and 65% of them were men. The total mean acute physiology and chronic health (APACHE) score of patients was 16.50 ± 4.87 ranging from 10 to 27. The main reasons for ICU admission among COPD patients included exacerbated COPD (*n* = 20), postoperative care (*n* = 23), stroke (*n* = 10), and diabetic ketoacidosis (*n* = 7) (Table 1).

**TABLE 1:** Demographic and clinical outcomes of COPD patients (*n* = 60).

Age (years)^a^	77.33 ± 8.29
Gender (*n*) ^b^	
Male	39 (65%)
Female	21 (35%)
APACHE score ^a^	16.50 ± 4.87
Indications of ICU admission (*n*) ^b^	
Exacerbated COPD	20 (33%)
Postoperative care	23 (38%)
Stroke	10 (17%)
Diabetic ketoacidosis	7 (12%)

Abbreviations: APACHE, Acute Physiology and Chronic Health Evaluation; COPD, chronic obstructive pulmonary disease; ICU, intensive care unit.

^a^ Continuous data are presented as mean ± standard deviation.

^b^ Categorical data are presented as frequency (%).

**FIGURE 1:**
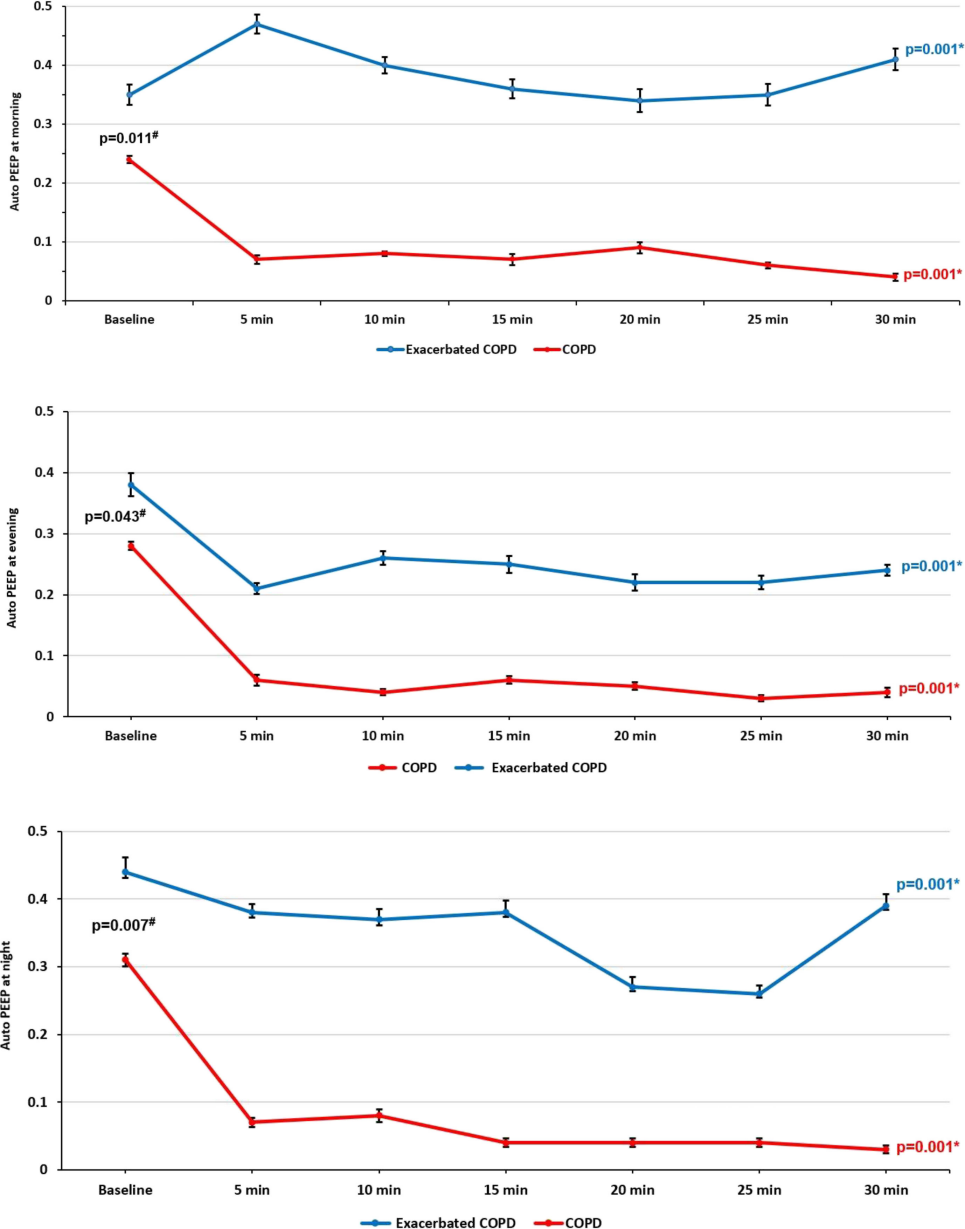
Mean changes in auto‐PEEP value of COPD and exacerbated COPD patients in the morning, evening, and night before (baseline) and after ATC activation, estimated by linear mixed model (LMM) analysis (reference: baseline). *p* values* (longitudinal change in mean auto‐PEEP calculated by LMM), *p* values^#^ (difference of bassline auto‐PEEP between COPD and exacerbated COPD).

In comparison with exacerbated COPD, baseline auto‐PEEP in COPD patients was significantly low in the morning (0.35 ± 0.16 vs. 0.24 ± 0.15, *p* = 0.011), evening (0.38 ± 0.19 vs. 0.28 ± 0.17, *p* = 0.043), and night (0.44 ± 0.15 vs. 0.31 ± 0.18, *p* = 0.007). As shown in Table 2, mean auto‐PEEP decreased significantly in all COPD patients after ATC activation in the morning, evening, and night (*p* = 0.001). Further analysis showed that the magnitude of auto‐PEEP decrease in COPD patients was notably greater than in exacerbated COPD patients (*p* = 0.001). Figure 1 provides more details and compares mean change in auto‐PEEP separately for COPD and exacerbated COPD patients before and after ATC activation in the morning, evening, and night.

**TABLE 2:** Change in mean auto‐PEEP before and after ATC activation in COPD patients.

Time	Before ATC	After ATC						
	Baseline	5 min	10 min	15 min	20 min	25 min	30 min	*p*
Morning	0.27 ± 0.16	0.21 ± 0.27	0.18 ± 0.25	0.16 ± 0.24	0.17 ± 0.27	0.16 ± 0.25	0.17 ± 0.27	0.001
Evening	0.32 ± 0.18	0.11 ± 0.16	0.12 ± 0.17	0.12 ± 0.17	0.10 ± 0.18	0.09 ± 0.16	0.11 ± 0.15	0.001
Night	0.35 ± 0.18	0.17 ± 0.22	0.18 ± 0.26	0.15 ± 0.26	0.11 ± 0.21	011. ± 0.18	0.14 ± 0.25	0.001

*Note:* Continuous data are presented as mean ± standard deviation. *p* value indicates mean change in auto‐PEEP calculated by linear mixed model analysis.

Abbreviations: ATC, automatic tube compensation; COPD, chronic obstructive pulmonary disease; PEEP, positive end‐expiratory pressure.

As described in the methodology section, age, gender, and type of COPD were inserted in the LMM as covariates to assess their effects on changes in mean auto‐PEEP in COPD patients. Table 3 represents obtained results, indicating that COPD exacerbation as a covariate was significantly associated with the changes in mean auto‐PEEP in the morning (*β* = −0.27, *p* = 0.001), evening (*β* = −0.16, *p* = 0.001), and night (*β* = −0.26, *p* = 0.001).

**TABLE 3:** The effect of presence or absence of exacerbation, gender, and age on auto‐PEEP.

Time	COPD exacerbation		Gender		Age
Morning	Yes = 0.36 ± 0.04 No = 0.09 ± 0.03	*β* = −0.27 *p* = 0.001	Male = 0.25 ± 0.02 Female = 0.20 ± 0.04	*β* = −0.044 *p* = 0.290	*β* = 0.0005 *p* = 0.830
Evening	Yes = 0.24 ± 0.02 No = 0.08 ± 0.01	*β* = −0.16 *p* = 0.001	Male = 0.18 ± 0.01 Female = 0.14 ± 0.02	*β* = −0.042 *p* = 0.070	*β* = 0.002 *p* = 0.14
Night	Yes = 0.35 ± 0.03 No = 0.09 ± 0.02	*β* = −0.26 *p* = 0.001	Male = 0.22 ± 0.02 Female = 0.21 ± 0.03	*β* = −0.009 *p* = 0.780	*β* = −0.0003 *p* = 0.860

*Note: p* value indicates effect of covariates on mean change in auto‐PEEP calculated by linear mixed model analysis.

Abbreviations: COPD, chronic obstructive pulmonary disease; PEEP, positive end‐expiratory pressure.

## 4 Discussion

ATC is a mode of weaning from MV that compensates for the drop of flow‐dependent pressure across an ETT over inspiration and expiration [14]. In individuals receiving MV, a significant relationship exists between expired‐carbon‐dioxide slope, resistance of the respiratory system, and auto‐PEEP, proposing that dynamic hyperinflation drives from sequential emptying of slow hypercapnic parts [15]. The important outcome of our investigation was that the activation of ATC mode can reduce auto‐PEEP in COPD patients but exacerbated form of COPD limited beneficial effects of activating ATC to reduce auto‐PEEP under MV. When ATC is activated, the pressure assist (P_ATC_) is continuously adjusted to the change in flow rate and flow‐dependent pressure decline during the ventilatory cycle, and therefore, ATC counteracts the ETT‐induced dynamic lung hyperinflation that resulted in decline in auto‐PEEP [14].

In normal pulmonary function, filled alveoli impose outward radial traction on collapsible airways and help hold the airways open over the exhalation. Alveolar destruction in COPD patients diminishes outward radial traction on the airways, lets the airway collapse, traps air in alveoli, and leads to increase in end‐expiratory lung volume (EELV) and decline in inspiratory capacity (IC) [16]. ATC was introduced to increase airway pressure through continuously estimating the drop of pressure across the ETT over inspiration and to decline airway pressure during expiration for maintaining alveolar pressure constantly [17]. At low levels of PEEP and/or high expiratory flow rates, airway pressure at the proximal end of the ETT is reduced to subatmospheric levels in order to succeed expiratory tube compensation completely. Through this mechanism, ATC compensates entirely for the tube‐related additional work of breathing [18, 19]. In patients who are endotracheally intubated, this has been linked to declined work of breathing, preservation of normal breathing pattern, better synchronization of patient with ventilator, and improved respiratory comfort [19]. Furthermore, adequate spontaneous breathing in ATC mode without any additional ventilatory support might be a worthwhile predictor of successful extubation in the late phase of weaning from MV, particularly in difficult‐to‐wean cases. However, the ATC mode is most effective at normal to high lung compliance, low airway resistance, and small ETT size [18, 19].

For many years, dynamic hyperinflation in ICU patients was considered a challenging problem for clinicians, especially in patients with either history of COPD or a chief complaint of COPD exacerbation [2, 20, 21]. In addition to flow limitation that plays a key role in the amount of auto‐PEEP, expiratory time/time constant, resistance of the respiratory system, and obesity are other most important variables affecting auto‐PEEP values. Armaganidis et al. reported that auto‐PEEP in 12 subjects with flow limitation was high in comparison to the 20 subjects without flow limitation [22]. It has been well reported that expiratory ATC significantly reduces ETT‐induced lung hyperinflation by facilitating lung emptying. In parallel with our findings, Haberthür et al. reported that compensation for expiratory ETT resistance by ATC increases tidal volume and decreases tracheal pressure to the level of external PEEP, reflecting reduced auto‐PEEP and, therefore, reduced dynamic hyperinflation [23].

Increased airflow obstruction and consequent dynamic hyperinflation in COPD with exacerbation lead to further air trapping, and patients adapt by assuming a rapid and shallow breathing pattern. This breathing pattern increases auto‐PEEP and results in further increase in the energy needed to start inspiration and increased work of breathing [24]. However, COPD patients with exacerbation could benefit from expiratory ATC but elevated airway resistance in such types of COPD will mostly exceed the resistive features of the ETT. Our findings and other analytical data indicate that such patients benefit less from expiratory ATC [23].

The present study has some potential limitations. The effects of partial tube obstruction on the performance of ATC did not assess in our patients. In the clinical setting, partial tube obstruction might happen due to secretions, tube kinking, or external compression of the ETT and reduces the cross‐sectional area of the ETT. Also, our findings are machine dependent and could well be different if we used another type of ventilator offering ATC mode.

## 5 Conclusion

Our findings in the current investigation showed that activating ATC in COPD patients could decrease the value of auto‐PEEP and markedly attenuate ETT‐related side effects. However, COPD patients with an exacerbation appear to benefit less from ATC activation for minimizing dynamic hyperinflation and auto‐PEEP.

## Author Contributions

*Conceptualization*: Omid Moradi Moghaddam, Shahab Mohammadi, Mohammad Niakan Lahiji, and Mohsen Sedighi. *Project administration*: Omid Moradi Moghaddam and Shahab Mohammadi. *Formal Analysis*: Mohsen Sedighi, Alireza Amanollahi, and Behrooz Zaman. *Writing – Original Draft Preparation*: Mohsen Sedighi, Omid Moradi Moghaddam, and Mahzad Alimian. *Writing – Review & Editing*: Mohsen Sedighi, Omid Moradi Moghaddam, and Mansoor Soltani.

## Ethics Statement

The protocol of the study was reviewed and approved by the Medical Research Ethic Committee at the Iran University of Medical Science (IR.IUMS.REC.1400.072) and written informed consent was given from all participants.

## Conflicts of Interest

The authors declare no conflicts of interest.

## Data Availability

The data that support the findings of this study are available from the corresponding author upon reasonable request.

## References

[1] M. J. Divo , B. R. Celli , B. Poblador‐Plou , et al., “Chronic Obstructive Pulmonary Disease (COPD) as a Disease of Early Aging: Evidence From the EpiChron Cohort,” PLoS ONE 13, no. 2 (2018): e0193143. 29470502 10.1371/journal.pone.0193143 PMC5823454

[2] D. E. O'donnell , S. M. Revill , and K. A. Webb , “Dynamic Hyperinflation and Exercise Intolerance in Chronic Obstructive Pulmonary Disease,” American Journal of Respiratory and Critical Care Medicine 164, no. 5 (2001): 770–777. 11549531 10.1164/ajrccm.164.5.2012122

[3] S. P. Patil , J. A. Krishnan , N. Lechtzin , and G. B. Diette , “In‐Hospital Mortality Following Acute Exacerbations of Chronic Obstructive Pulmonary Disease,” Archives of Internal Medicine 163, no. 10 (2003): 1180–1186. 12767954 10.1001/archinte.163.10.1180

[4] P. Almagro , B. Barreiro , A. O. de Echagüen , et al., “Risk Factors for Hospital Readmission in Patients With Chronic Obstructive Pulmonary Disease,” Respiration 73, no. 3 (2006): 311–317. 16155352 10.1159/000088092

[5] J. Garcia‐Aymerich , E. Monso , R. M. Marrades , et al., “Risk Factors for Hospitalization for a Chronic Obstructive Pulmonary Disease Exacerbation: EFRAM Study,” American Journal of Respiratory and Critical Care Medicine 164, no. 6 (2001): 1002–1007. 11587986 10.1164/ajrccm.164.6.2006012

[6] F. Renom , A. Yáñez , M. Garau , et al., “Prognosis of COPD Patients Requiring Frequent Hospitalization: Role of Airway Infection,” Respiratory Medicine 104, no. 6 (2010): 840–848. 20106648 10.1016/j.rmed.2009.12.010

[7] C. Sasannejad , E. Ely , and S. Lahiri , “Long‐Term Cognitive Impairment After Acute Respiratory Distress Syndrome: A Review of Clinical Impact and Pathophysiological Mechanisms,” Critical Care 23, no. 1 (2019): 1–12. 31718695 10.1186/s13054-019-2626-z PMC6852966

[8] L. Blanch , F. Bernabé , and U. Lucangelo , “Measurement of Air Trapping, Intrinsic Positive End‐Expiratory Pressure, and Dynamic Hyperinflation in Mechanically Ventilated Patients,” Respiratory Care 50, no. 1 (2005): 110–124. 15636649

[9] A. D. T. Force , V. Ranieri , G. Rubenfeld , et al., “Acute Respiratory Distress Syndrome,” Journal of the American Medical Association 307, no. 23 (2012): 2526–2533. 22797452 10.1001/jama.2012.5669

[10] V. Antonaglia , M. Ferluga , G. Capitanio , et al., “Respiratory Mechanics in COPD Patients Who Failed Non‐Invasive Ventilation: Role of Intrinsic PEEP,” Respiratory Physiology & Neurobiology 184, no. 1 (2012): 35–40. 22877584 10.1016/j.resp.2012.07.009

[11] L. Gattinoni , F. Collino , G. Maiolo , et al., “Positive End‐Expiratory Pressure: How to Set It at the Individual Level,” Annals of Translational Medicine 5, no. 14 (2017): 288. 28828363 10.21037/atm.2017.06.64 PMC5537121

[12] J. D. Cohen , M. Shapiro , E. Grozovski , and P. Singer , “Automatic Tube Compensation‐Assisted Respiratory Rate to Tidal Volume Ratio Improves the Prediction of Weaning Outcome,” Chest 122, no. 3 (2002): 980–984. 12226043 10.1378/chest.122.3.980

[13] J. Oto , H. Imanaka , E. Nakataki , R. Ono , and M. Nishimura , “Potential Inadequacy of Automatic Tube Compensation to Decrease Inspiratory Work Load After at Least 48 Hours of Endotracheal Tube use in the Clinical Setting,” Respiratory Care 57, no. 5 (2012): 697–703. 22153219 10.4187/respcare.01380

[14] S. Elsasser , J. Guttmann , R. Stocker , G. Mols , H.‐J. Priebe , and C. Haberthür , “Accuracy of Automatic Tube Compensation in New‐Generation Mechanical Ventilators,” Critical Care Medicine 31, no. 11 (2003): 2619–2626. 14605533 10.1097/01.CCM.0000094224.78718.2A

[15] B. Babik , Z. Csorba , D. Czövek , P. N. Mayr , G. Bogáts , and F. Peták , “Effects of Respiratory Mechanics on the Capnogram Phases: Importance of Dynamic Compliance of the Respiratory System,” Critical Care 16, no. 5 (2012): 1–10. 10.1186/cc11659 PMC3682277 23031408

[16] D. E. O'Donnell , J. C. Bertley , L. Chau , and K. A. Webb , “Qualitative Aspects of Exertional Breathlessness in Chronic Airflow Limitation: Pathophysiologic Mechanisms,” American Journal of Respiratory and Critical Care Medicine 155, no. 1 (1997): 109–115. 9001298 10.1164/ajrccm.155.1.9001298

[17] I. Grossbach , L. Chlan , and M. F. Tracy , “Overview of Mechanical Ventilatory Support and Management of Patient‐and Ventilator‐Related Responses,” Critical Care Nurse 31, no. 3 (2001): 30–44. 10.4037/ccn2011595 21632592

[18] C. Haberthür , G. Mols , S. Elsasser , R. Bingisser , R. Stocker , and J. Guttmann , “Extubation After Breathing Trials With Automatic Tube Compensation, T‐Tube, or Pressure Support Ventilation,” Acta Anaesthesiologica Scandinavica 46, no. 8 (2002): 973–979. 12190798 10.1034/j.1399-6576.2002.460808.x

[19] G. Mols , E. Rohr , A. Benzing , C. Haberthür , K. Geiger , and J. Guttmann , “Breathing Pattern Associated With Respiratory Comfort During Automatic Tube Compensation and Pressure Support Ventilation in Normal Subjects,” Acta Anaesthesiologica Scandinavica 44, no. 3 (2000): 223–230. 10714832 10.1034/j.1399-6576.2000.440302.x

[20] P. Calverley and N. Koulouris , “Flow Limitation and Dynamic Hyperinflation: Key Concepts in Modern Respiratory Physiology,” The European Respiratory Journal 25, no. 1 (2005): 186–199. 15640341 10.1183/09031936.04.00113204

[21] D. E. O'Donnell and K. A. Webb , “The Major Limitation to Exercise Performance in COPD Is Dynamic Hyperinflation,” Journal of Applied Physiology 105, no. 2 (2008): 753–755. 18678624 10.1152/japplphysiol.90336.2008b

[22] A. Armaganidis , K. Stavrakaki‐Kallergi , A. Koutsoukou , A. Lymberis , J. Milic‐Emili , and C. Roussos , “Intrinsic Positive end‐Expiratory Pressure in Mechanically Ventilated Patients With and Without Tidal Expiratory Flow Limitation,” Critical Care Medicine 28, no. 12 (2000): 3837–3842. 11153623 10.1097/00003246-200012000-00015

[23] C. Haberthür , A. Mehlig , J. F. Stover , et al., “Expiratory Automatic Endotracheal Tube Compensation Reduces Dynamic Hyperinflation in a Physical Lung Model,” Critical Care 13, no. 1 (2009): R4. 19166607 10.1186/cc7693 PMC2688116

[24] R. M. Reddy and K. K. Guntupalli , “Review of Ventilatory Techniques to Optimize Mechanical Ventilation in Acute Exacerbation of Chronic Obstructive Pulmonary Disease,” International Journal of Chronic Obstructive Pulmonary Disease 2, no. 4 (2007): 441–452. 18268918 PMC2699957

